# P. GINGIVALIS-LPS PROMOTES AD/ADRD VIA CASPASE-4-MEDIATED NONCANONICAL INFLAMMASOME PATHWAY

**DOI:** 10.1093/geroni/igae098.3082

**Published:** 2024-12-31

**Authors:** Ambika Verma, Gohar Azhar, Pankaj Patyal, Xiaomin Zhang, Jeanne Y Wei

**Affiliations:** University of Arkansas for Medical Sciences, Little Rock, Arkansas, United States; University of Arkansas for Medical Sciences, Little Rock, Arkansas, United States; University of Arkansas for Medical Sciences, Little Rock, Arkansas, United States; University of Arkansas for Medical Sciences, Little Rock, Arkansas, United States; University of Arkansas for Medical Sciences, Little Rock, Arkansas, United States

## Abstract

Alzheimer’s disease (AD) and related dementias (ADRD) are significant contributors to illness and death in the older adult population worldwide. Chronic periodontitis and its keystone pathogen, Porphyromonas gingivalis, have increasingly been linked with AD/ADRD. P. gingivalis releases various virulence factors, including lipopolysaccharide (LPS), which have been shown to trigger systemic inflammatory events responsible for neuroinflammation and AD/ADRD. Caspase-4 directly senses and binds cytosolic LPS, promoting its oligomerization and activation, leading to the formation of the non-canonical inflammasome. However, the role of caspase-4 in P. gingivalis- LPS mediated AD-related neuropathology remains poorly defined. We hypothesized that P. gingivalis- LPS may be responsible for neuroinflammation via activation of caspase-4 mediated non-canonical inflammasome pathway through activation of its downstream NOD-like receptor family pyrin domain containing 3 (NLRP3) and caspase-1 inflammasome dependent cell signaling. Our overall hypothesis is exploring the cell signaling pathway by which P. gingivalis- LPS are able to affect the microglial/neuronal cells via activating caspase-4, that might be responsible for neuroinflammation and lead to AD/ADRD. Our results suggest that P. gingivalis- LPS activated caspase-4 and cleaved gasdermin D (GSDMD) to form the GSDMD pores to trigger pyroptosis, a form of programmed lytic cell death. The GSDMD pores in the plasma membrane also promoted K+ influx, sensed by the NLRP3 inflammasome to activate caspase-1, resulting in IL-1β maturation and secretion, as well as pyroptosis. This P. gingivalis- LPS induced caspase-4-mediated NLRP3/caspase-1 activation and associated IL-1β secretion, promotes neuroinflammatory environment that drives AD/ADRD pathogenesis.

